# Cement Remnants Thickness After Polishing With Tungsten, Diamond, and Arkansas Bur Using Composite Customized Lingual Brackets

**DOI:** 10.1002/cre2.70302

**Published:** 2026-01-29

**Authors:** Javier Flores‐Fraile, Alba Belanche Monterde, Francesca Gorassini, Álvaro Zubizarreta‐Macho, Artak Heboyan, Cosimo Galletti

**Affiliations:** ^1^ Department of Surgery Faculty of Medicine University of Salamanca Salamanca Spain; ^2^ Department of Dental Research Cell, Dr. D. Y. Patil Dental College & Hospital Dr. D. Y. Patil Vidyapeeth (Deemed to be University) Pimpri Pune India; ^3^ FiDent, Centro Medico Odontoiatrico Reggio di Calabria Italy; ^4^ Faculty of Dentistry Alfonso X el Sabio University Madrid Spain; ^5^ Department of Research Analytics, Saveetha Institute of Medical and Technical Sciences, Saveetha Dental College and Hospitals Saveetha University Chennai India; ^6^ Department of Prosthodontics, Faculty of Stomatology Yerevan State Medical University after Mkhitar Heratsi Yerevan Armenia; ^7^ Department of Prosthodontics, School of Dentistry Tehran University of Medical Sciences Tehran Iran; ^8^ School of Medicine and Surgery University of Enna Enna Italy

**Keywords:** adhesion, bracket, burs, composite

## Abstract

**Introduction:**

There is no current evidence in the literature that clearly guides clinicians in selecting the most effective polishing protocol in lingual orthodontics. This study aimed to compare the reduction of adhesive remnants after polishing customized lingual composite brackets using tungsten carbide, fine diamond, and Arkansas burs.

**Methods:**

A total of 504 extracted teeth were included and randomly assigned to three groups according to bur type (*n* = 168). Brackets were bonded and debonded following a standardized protocol, and digital scans were obtained before and after polishing. Each bur was tested at both low speed (contra‐angle) and high speed (turbine).

**Results:**

Tungsten carbide burs produced the greatest reduction in cement thickness under both rotary conditions. At low speed, the mean Pre–Post thickness differences were 0.64 mm (TUN), 0.31 mm (ARK), and 0.37 mm (DIA). At high speed, differences were 0.45 mm (TUN), 0.39 mm (ARK), and 0.41 mm (DIA). Statistically significant differences were found between the tungsten carbide group and both the Arkansas and diamond groups (*p* < 0.005), with no differences between the latter two.

**Conclusion:**

Tungsten carbide burs removed significantly more adhesive than Arkansas and diamond burs, regardless of rotary speed. These findings support clinical decision‐making by helping optimize polishing protocols in lingual orthodontics.

## 1 Introduction

Polishing has been studied after braces and attaches adhesion in order to find the best polishing protocol that allows to maintain enamel integrity. It has been observed that two‐step polishing allows a good surface preservation after aligner treatment (Khorn et al. 2024). Another esthetic orthodontic option developed in the 1970s in the United States is the lingual multibracket appliance. It is the only fully invisible orthodontic option because no appliance is bonded to the vestibular surface of the teeth. However, lingual braces present biomechanical challenges due to the reduced inter‐bracket distance, which results in greater force expression with minimal activation. Also, it needs expansion compensation due to the lingual application of forces (George and Hirani 2013). More recently, lingual braces were customized in order to avoid mechanical affection of the lingual surfaces into orthodontics finalization. Customized lingual braces are disposable by adding composite into the brace base or individualizing the metal base. Lingual braces can also be self‐ligating common ligating, condition that can also affect biomechanics (Scuzzo et al. 2010). Furthermore, polishing has been widely studied after orthodontic debonding in order to avoid side effects. It has been observed that diamond polishing had effects on the enamel reducing its roughness after vestibular orthodontics debonding (Fahimi et al. 2024). Whereas, lingual and vestibular surfaces of teeth have lots of differences due to inter‐ and intra‐individual variations lingual surfaces have in between (Botzenhart et al. 2012). Sear bond strength (SBS) has also been studied in lingual appliances because of the low evidence literature has on lingual adhesion. In addition, the lingual enamel morphology has been analyzed using optical coherence tomography and scanning electron microscope before and after surfaces conditioning. Sandblasting was not useful in increasing SBS when adhering lingual braces (Lopes et al. 2021). Etching and adhesives were also evaluated with lingual customized braces, and etching was highly recommended even using self‐etch and self‐adhesive resins in order to reduce bonding failures (Mavreas et al. 2018). However, a meta‐analysis (Namdari et al. 2021) did not found statistical differences between conventional and self‐etch primers in lingual braces debonding (Namdari et al. 2021). Enamel roughness is increased after adhesive removal which is associated with bacterial adhesion and caries. That is why the tool of polishing is studied in orthodontics by confocal laser microscopy. Tungsten carbide burs have previously been shown to highly increase enamel roughness in comparison to adhesive residue removers (Janiszewska‐Olszowska et al. 2016). Studies whose aim is measuring the volume of cement remains after braces debonding use a novel methodology called morphometry based on standard tessellation language (STL) software (Belanche Monterde et al. 2021a, 2021b). Moreover, effects of polishing and debonding have also been studied on zirconia surfaces comparing different polishing bur protocols. It was observed that tungsten carbide burs (TC) at low speed and fine diamond bur at a high speed attach to zirconia surface roughness and to flexural strength of this material (Babaee Hemmati et al. 2022).

The aim of the present study is to compare the volume and the area of the adhesive remnants after customized lingual braces debonding and polishing with three different polishing burs.

## 2 Methodology

### 2.1 Trial Design

This research is an experimental in vitro study performed in Salamanca University (Department of Surgery, Salamanca, Spain) between 2021 and 2024. In the trial area, thickness of the cement remains after debonding lingual ALIAS system braces was measured. For that, STLs were taken previous polishing and after the protocol. This study was carried out in accordance with the criteria defined in the regulations of the German Ethics Committee on the use of organic tissues in medical research. The patients gave up the extracted teeth voluntarily after signing the Informed Consent.

### 2.2 Trial Justification and Risk Assessment

Due to the differences and characteristics of lingual multibrackets appliances and lingual enamel surfaces, it seems crucial leading a safe and effective debonding and polishing. Polishing in lingual braces has been low studied, and there are not lots of methodologies for measuring cement remains after polishing that allows clinicians choose the best bur or protocol. In addition, lingual polishing might be more complex than vestibular because of the difficulties in access and tongue. Also, it has been shown that enamel microanatomy in lingual is different from that in vestibular, which could lead to adhesion failures, so in braces debonding (Babaee Hemmati et al. 2022; Raticová et al. 2024; Moradi et al. 2018). In the current evidence, most of the articles that investigate polishing and debonding are interested in roughness, but only a few articles talk about the effectiveness of polishing. So, that is the reason why the present in vitro trial focus on measuring the amount of remnants. Nowadays, “polishing seems to be safe with the most burs used for polishing in orthodontics which are tungsten carbide bur, Arkansas bur and fine diamond bur” (Babaee Hemmati et al. 2022). In addition, the ALIAS system lingual braces was used because of being composite base customized and self‐ligating, so it was understood it could lead to higher composite remnants than another customized braces (Takemoto et al. 2009; Maizeray et al. 2021).

### 2.3 Sample Size and Inclusion Criteria

The extracted teeth were selected, meeting some inclusion criteria: extracted teeth due to periodontitis or orthodontic reasons, teeth with intact coronas and anatomy, and teeth with integral enamel surfaces without previous polishing or restorations. Teeth extracted by endodontics, anomalous teeth, and restored teeth or carious affected were totally excluded. A preliminary trial with 13 teeth was previously made in order to establish the methodology and avoid errors. After that a total of 504 were used, 168 for each bur group.

### 2.4 Sample Processes

Teeth were randomly selected for each group with a statistical software (Epidat 4.1, Galicia, Spain). All groups were polished using both low‐speed (contra‐angle) and high‐speed (turbine) handpieces (Moradi et al. 2018). This was done to compare the efficiency of each bur under both rotary systems.

− Group 1: polishing tungsten carbide bur (TUG) (H23VIP.204.016, Komet, Lemgo, Alemania).
− Group 2: polishing Arkansas bur (ARK) (07486K0, Komet, Lemgo, Alemania).
− Group 3: fine polishing diamond bur (DIA) (32189K2, Komet, Lemgo, Alemania).

### 2.5 Interventions

The teeth were embedded in 36 epoxy resin models (Ref. 20‐8130‐128, EpoxiCure, Buehler, IL, USA) simulating a dental arch. The study models consisted of 14 teeth (central incisors, lateral incisors, canines, first and second premolars, and first and second molars). Initially, the lingual‐palatal surface of the teeth was preconditioned with 37% orthophosphoric acid (Ortho Solo, Ormco Corporation, CA, USA). Subsequently, a primer adhesive (Unitek Transbond XT, 3 M ESPE, Saint Paul, MN, USA) was applied with a microbrush (Plus slim, Microbrush Inter., Grafton, MA, USA). After this, the lingual brackets (ALIASTM, Ormco, California, USA) were bonded directly to the lingual surface of the teeth using a bracket bonding forceps (IX850, Ixion Instruments, DB Orthodontics, Keighley, United Kingdom). Cementation of the lingual brackets was performed from first to first maxillary molars in the study model using a composite resin cement (Transbond XT, 3 M ESPE, Saint Paul, MN, USA). The brackets were then debonded with a specialized bracket debonding pliers (IX827, Ixion Instruments, DB Orthodontics, Keighley, UK). Then, all of them were subjected to a digital impression using an intraoral scan (True Definition, 3 M ESPE, Saint Paul, MN, USA) acquiring a STL image (STL1). Finally, the lingual surfaces were polished using the visual method of detecting cement remnants. The teeth were randomly assigned to the study groups differing in the polishing procedure all performed with irrigation. Each polishing protocol was performed twice: first with a low‐speed contra‐angle and then with a high‐speed turbine. This methodological detail aligns with the results presented for both rotary systems (low‐speed and turbine) in Tables 2, 3, 4. Once the operator had finished polishing the surfaces, another digital impression was made after polishing (STL2).

### 2.6 Measurement Methodology

The STLs obtained were uploaded to the 3D software (Dolphin Imaging 64‐bit, Dolphin Imaging & Management Solutions, Chatsworth, California) in a new folder created for the study. All STLs of the full arch were segmented tooth by tooth with a sculpting tool available in the 3D software. This was done to reduce the maximum possible error during the STL overlay process. STL 1‐2 were aligned to perform the measurements corresponding to the study. For this, an initial superimposition was carried out by points. This was done by placing three points in the same location in three‐dimensional space. After that, to increase the precision of the superposition, another tool available in Dolphing Imaging 3D (Dolphin Imaging 64‐bit, Dolphin Imaging & Management Solutions, Chatsworth, California) called ColorMap was used, which allows detecting minimum distances between the superpositions and showing them as color changes from red to blue. ColorMap allows differences in distances greater than 1 mm, showing them in red and showing distances close to 1 mm in blue. The overlays were adjusted until the buccal surfaces were completely blue; however, the lingual surfaces showed red areas corresponding to the changes produced during polishing. The superpositions were visualized in different planes to evaluate possible errors. Linear cement measurements were evaluated by a 2D linear measurement tool available in Dolphing Imaging 3D (Dolphin Imaging 64‐bit, Dolphin Imaging & Management Solutions, Chatsworth, California). The cement remnant area (mm²) was calculated from the STL superimposition by selecting the region corresponding to the adhesive residue in both the pre‐polishing and post‐polishing scans. The 3D software automatically quantified the enclosed surface, allowing estimation of the reduction in cement area (pre–post difference) for each bur and rotary system. With it, the greatest distances of width and height in frontal view and thickness in sagittal view of remaining cement in the different study groups were measured. This measurement methodology was previously analyzed with Gage R&R statistical analysis to study if it was repeatable and reproducible. The measurement technique by superimposing scanners with the Dolphing Imaging 3D software proved to be a repeatable and reproducible technique (Aksoz et al. 2025).

### 2.7 Statistical Analysis

The data were collected into Excel sheets (Excel Microsoft). The statistical analysis was done with SAS v9.4 (SAS Institute Inc., NC, USA). The statistical limit was set to *p* < 0.005. *p*‐values have been adjusted by the Bonferroni correction for multiplicity of contrasts. Due to the lack of normality of the data (Shapiro‐Wilk test), the non‐parametric contrasts of Mann–Whitney–Wilcoxon or Kruskal–Wallis were applied.

## 3 Results

### 3.1 Processed Data

**Figure 1:**
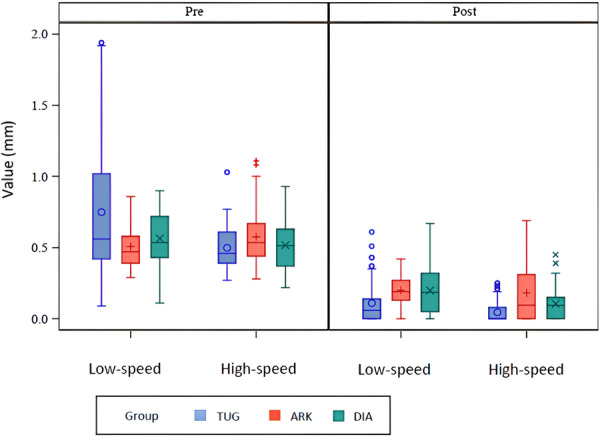
Boxplots illustrating pre‐ and postoperative cement thickness for each polishing bur (Tungsten carbide, Arkansas, Diamond) under low‐speed polishing conditions. Greater differences indicate more effective cement removal.

The descriptive analysis of the width and height using the three burs and low hand piece system was presented in Table 1 and Figure 1.

**Table 1:** Pre‐ and postoperative descriptive measurements (width and height) of cement remnants for the three polishing burs (Tungsten carbide, Arkansas, and Diamond) using a low‐speed contra‐angle handpiece. Values are expressed as mean, standard deviation, median, quartiles, minimum, and maximum. Each group includes 168 teeth.

			**Contra‐angle values**								
			N	Mean	Std	Min	Q1	Median	Q3	Max	NMiss
TUN	Width	Pre	84	4.03	1.07	1.16	3.52	3.87	4.83	5.79	0
		Post	84	0.46	0.51	0.00	0.00	0.40	0.77	1.97	0
	Height	Pre	84	3.41	0.92	0.79	2.80	3.61	4.10	4.85	0
		Post	84	0.61	0.74	0.00	0.00	0.44	0.91	2.65	0
ARK	Width	Pre	84	4.49	0.97	3.27	3.88	4.12	5.16	7.48	0
		Post	84	2.32	1.53	0.00	0.89	2.22	3.81	5.23	0
	Height	Pre	84	3.53	0.67	2.07	2.99	3.40	4.02	4.74	0
		Post	84	1.24	1.01	0.00	0.47	0.90	2.12	3.82	0
DIAM	Width	Pre	84	4.00	1.00	1.49	3.33	3.85	4.53	6.09	0
		Post	84	1.31	1.13	0.00	0.48	0.94	2.19	3.98	0
	Height	Pre	84	3.38	0.94	1.35	2.95	3.27	3.88	5.51	0
		Post	84	1.14	1.02	0.00	0.42	0.91	1.82	4.13	0

**Figure 2:**
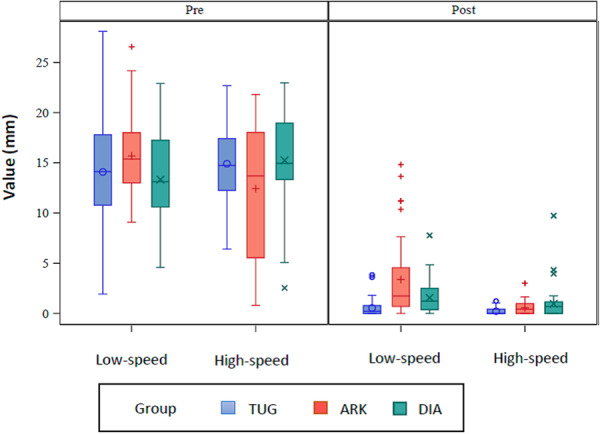
Comparison of estimated pre‐ and postoperative cement areas according to the polishing bur and the rotary system (low‐speed vs high‐speed). Decreases in area indicate greater cement removal efficiency.

In Table 2 and Figure 2, descriptive data of each group were presented using turbine.

**Table 2:** Pre‐ and postoperative descriptive measurements (width and height) of cement remnants for the three polishing burs (Tungsten carbide, Arkansas, Diamond) using a high‐speed turbine handpiece. Values include mean, standard deviation, median, quartiles, minimum, and maximum for 168 samples per group.

			Turbine values								
			N	Mean	Std	Min	Q1	Median	Q3	Max	NMiss
TUN	Width	Pre	84	4.41	0.97	2.24	3.67	4.07	5.52	6.21	0
		Post	84	0.41	0.65	0.00	0.00	0.00	0.64	2.90	0
	Height	Pre	84	3.41	0.61	1.76	2.98	3.42	3.93	4.56	0
		Post	84	0.28	0.47	0.00	0.00	0.00	0.46	2.42	0
ARK	Width	Pre	84	4.03	1.38	0.84	3.44	4.22	5.13	5.91	0
		Post	84	0.86	0.98	0.00	0.00	0.57	1.48	3.86	0
	Height	Pre	84	3.02	1.13	0.38	2.58	3.27	3.78	4.63	0
		Post	84	0.47	0.47	0.00	0.00	0.44	0.74	1.87	0
DIAM	Width	Pre	84	3.92	1.01	1.47	3.27	3.89	4.50	6.01	0
		Post	84	1.13	1.25	0.00	0.00	0.81	1.62	4.95	0
	Height	Pre	84	3.95	1.00	1.00	3.47	4.02	4.44	6.42	0
		Post	84	0.64	0.70	0.00	0.00	0.45	0.89	2.68	0

**Figure 3:**
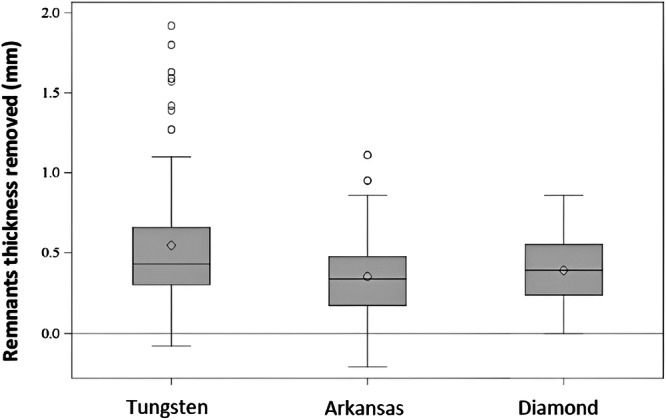
Boxplots showing pre‐ and postoperative cement thickness differences for each polishing bur. Larger pre–post values indicate greater polishing efficiency. Finally, for each tooth, the Pre‐Post difference in millimeters was calculated in order to estimate the cement remains area. The descriptive data of the area are presented in Table 4 and Table 5.

After that, a descriptive statistical analysis was done for thickness measurements shown in Table 3 and Figure 3.

− Difference thickness = Thickness mm pre ‐ Thickness mm post
− Difference area = Area mm^2^ Pre ‐ Area mm^2^ post

**Table 3:** Pre‐ and postoperative thickness measurements of cement remnants for the three polishing burs under both low‐speed and high‐speed conditions. Results include mean, standard deviation, median, quartiles, minimum, and maximum values.

		Cement thickness					
		Pre			Post		
		N	Mean	Std	N	Mean	Std
Contra‐angle	TUN	84	0.75	0.47	84	0.11	0.14
	ARK	84	0.51	0.14	84	0.20	0.10
	DIA	84	0.56	0.21	84	0.20	0.16
Turbine	TUN	84	0.50	0.16	84	0.05	0.07
	ARK	84	0.58	0.20	84	0.18	0.21
	DIAM	84	0.52	0.17	84	0.11	0.10

### 3.2 Statistical Analysis and Trends

The average of the pre‐post differences in thickness after polishing with Contra Angle and Tungsten bur was 0.64 mm (std = 0.5). The mean with Contraangle‐Arkansas bur and Contraangle‐Diamante were 0.31 mm (std = 0.15) and 0.37 mm (std = 0.21), respectively. The mean pre‐post thickness differences in the Turbine group were: 0.45 mm (std = 0.18) for Tungsten, 0.39 mm (std = 0.3) for Arkansas, and 0.41 mm (std = 0.19) for diamond groups. The data were presented in Table 5 and Figure 3. In all the three groups, statistically significant differences were detected between the observed distribution and the normal distribution. Thus, the non‐parametric Kruskal–Wallis test was performed to compare the values of the pre‐post thickness difference. There are statistically significant differences (chi‐squared = 25.87, *p*‐value < 0.0001) between drills in the difference in pre‐postoperative thickness. This means that cement remnants are more removed in the Tungsten bur group. Whereas, there were no differences between Arkansas and diamond bur in terms of efficiency.

**Table 4:** Estimated cement area (mm²) before and after polishing for each bur type (Tungsten carbide, Arkansas, Diamond) under both rotary systems. The difference (pre–post) represents the amount of cement removed.

		Cement remains area					
		Pre			Post		
		N	Mean	Std	N	Mean	Std
Contra‐angle	TUN	84	14.08	5.71	84	0.55	0.88
	ARK	84	15.68	3.78	84	3.37	3.93
	DIAM	84	13.32	4.30	84	1.57	1.60
Turbine	TUN	84	14.90	3.63	84	0.22	0.33
	ARK	84	12.42	6.34	84	0.57	0.65
	DIAM	84	15.24	4.51	84	0.97	1.66

**Table 5:** Comparative analysis of pre‐ and postoperative cement thickness across bur groups. Includes statistical comparisons to identify differences in polishing efficiency among burs.

	Thickness of cement remains removed (mm)								
	N	Mean	Std	Min	Q1	Median	Q3	Max	NMiss
Tungsten	168	0.55	0.39	−0.08	0.30	0.43	0.66	1.92	0
Arkansas	168	0.35	0.24	−0.21	0.18	0.34	0.48	1.11	0
Diamond	168	0.39	0.20	0.00	0.24	0.39	0.55	0.86	0

## 4 Discussion

The quantification of adhesive remnants, rather than roughness alone, provides clinically relevant insights into the efficiency of polishing protocols in lingual orthodontics. In the present in vitro study, it was observed that tungsten carbide bur has better results in terms of thickness cement reduction after lingual braces. The tungsten carbide burs used were specially chosen because they are special for lingual orthodontics and allow damaging the enamel.

Orthodontics is a dentistry field which focuses on esthetics and occlusion but some discomfort has been observed in patients due to visibility of braces since its beginning. Due to that, esthetics braces were created, also bonded in vestibular surfaces of teeth. Whereas, it led to disappointment of patients and clinicians because it was still visible and also because of the frictional and breaking problems they had (Shibani et al. 2020). More recently, clear aligners were created with biomechanical differences and better esthetic outcomes in contrast with vestibular braces. However, it was observed that also aligners are visible because of the attachments bonded on vestibular enamel and the own plastic material that applies forces (Thai et al. 2020). Lingual braces remain the only completely invisible fixed orthodontic option. But, higher discomfort has been related by tongue hurt (Moradi et al. 2018). Another concern in orthodontics is with spot lesion consequent to adhesion. It has been studied that lingual orthodontics seems to reduce these initial caries lesion due to the tongue action that avoids plaque adhesion. However, the available evidence remains of low quality (Paula et al. 2017).

In addition, polishing protocols and burs after brackets debonding have been studied but focused on final roughness or SBS (Sayahpour et al. 2025; Alzaid et al. 2024). Adhesive remnant index (ARI) was very common in the literature in order to evaluate cement remnants. But, this index is observational, so it does not allow to obtain a real measurement to compare the data. It was observed that two steps polishing using tungsten carbide burs followed by smoozie polishers allows to significantly obtain enamel surfaces with less roughness than using tungsten carbide burs alone. But, two steps polishing was high time consuming compared to one step protocol (Sayahpour et al. 2025). In the current in vitro trial, a one‐step polishing with only polishing burs was performed in order to compare the main reduction of cement remnants because roughness evaluation was not the purpose of the study. Profilometers were also used in order to measure enamel roughness after polishing. It was observed that polishing with electric hand pieces got higher roughness values than high speed hand piece. So, it seems that electric hand pieces are not efficient compared to turbine (Yousry et al. 2024). Another study agrees with the fact that two‐step polishing after debracking allows better roughness results than using tungsten carbide bur alone. The second polishing step was Soft‐Lex disc. It was shown that polishing pastes are not needed because it did not obtain statistical differences in roughness parameters. The cement used for bonding significantly affected to polishing. Transbond XT required more time waste in polishing than Fuji cement but Transbond XT obtained less cement remnants (Křivková et al. 2023). The convexity upon enamel surfaces also affects the polishing and could damage the enamel. Also, commonly, the braces are bonded in the most convex part of the surface. Premolars also have a higher convexity compared to incisors in its vestibular surface (Pallarés‐Serrano et al. 2024). However, lingual enamel surfaces have totally heterogeneous anatomies having even inter‐individual differences. Lingual convexity is higher, and commonly braces are placed more apical than in vestibular in order to be closely bonded to resistance center of teeth (Babaee Hemmati et al. 2022; Raticová et al. 2024; Moradi et al. 2018). On the other hand, studies that compare polishing between burs use magnifying loupes and compare naked eye with augmentation. Tungsten carbide bur with loupes got the lowest roughness values when compared with Arkansas burs with loupes. Also with naked eye, tungsten carbide bur obtained fewer roughness values than white stones with magnification (Thawaba et al. 2022). Diamond burs seem to be the most time‐wasting with no significant benefits. In addition, Soft‐Lex discs even allow less enamel roughness, it also produce higher temperature on the surface. So, it steams unclear the best polishing protocol and some author recommends to individualize the protocol in dependence to the clinical case (Almudhi et al. 2023; Yassaei et al. 2023; Gorassini et al. 2024; Alamri and Al‐Dulaijan 2025; D'Andrea et al. 2021).

Within the limitations of the present study, the best bur for totally eliminating the cement remains was the tungsten carbide bur selected specially for lingual surfaces. No enamel damage was apparently observed in the scans. Therefore, it must be taken into account that there are some limitations, as difficult access to the lingual surfaces, lower clinician vision, and the amount of saliva.

## 5 Conclusion

The tungsten carbide bur reduced significantly more cement than the diamond and Arkansas burs. However, more evidence and in vivo studies are needed in this field.

## Author Contributions

Conceptualization: Alba Belanche Monterde and ÁlvaroZubizarreta‐Macho. Methodology: Alba Belanche Monterde and Javier Flores‐Fraile. Supervision: ÁlvaroZubizarreta‐Macho, Artak Heboyan and JavierFlores‐Fraile. Writing and original draft preparation: Alba Belanche Monterde. Writing—review and editing: Javier Flores‐Fraile, Artak Heboyan and Álvaro Zubizarreta‐Macho. Supervision and project administration: JavierFlores‐Fraile. All authors have read and agreed to the published version of the manuscript.

## Funding

The authors received no specific funding for this work.

## Ethics Statement

The authors have nothing to report.

## Consent

The authors have nothing to report.

## Conflicts of Interest

The authors declare no conflicts of interest.

## Data Availability

The original contributions presented in this study are included in the article. Further inquiries can be directed to the corresponding author.
